# Supplementary material for: Causal interplay between lactose intolerance and gut microbiota: a combined bidirectional Mendelian randomization and in vivo validation study
Source: Front Nutr. 2026 Jun 1;13:1803337. doi: 10.3389/fnut.2026.1803337 (PMC13265576; doi:10.3389/fnut.2026.1803337)
Supplement: Supplementary file 1 [file Data_Sheet_1.zip › supplementary materials/Forward/leave one out cross validation/ebi-a-GCST90027637.finngen_R12_E4_LACTONAS.pdf]

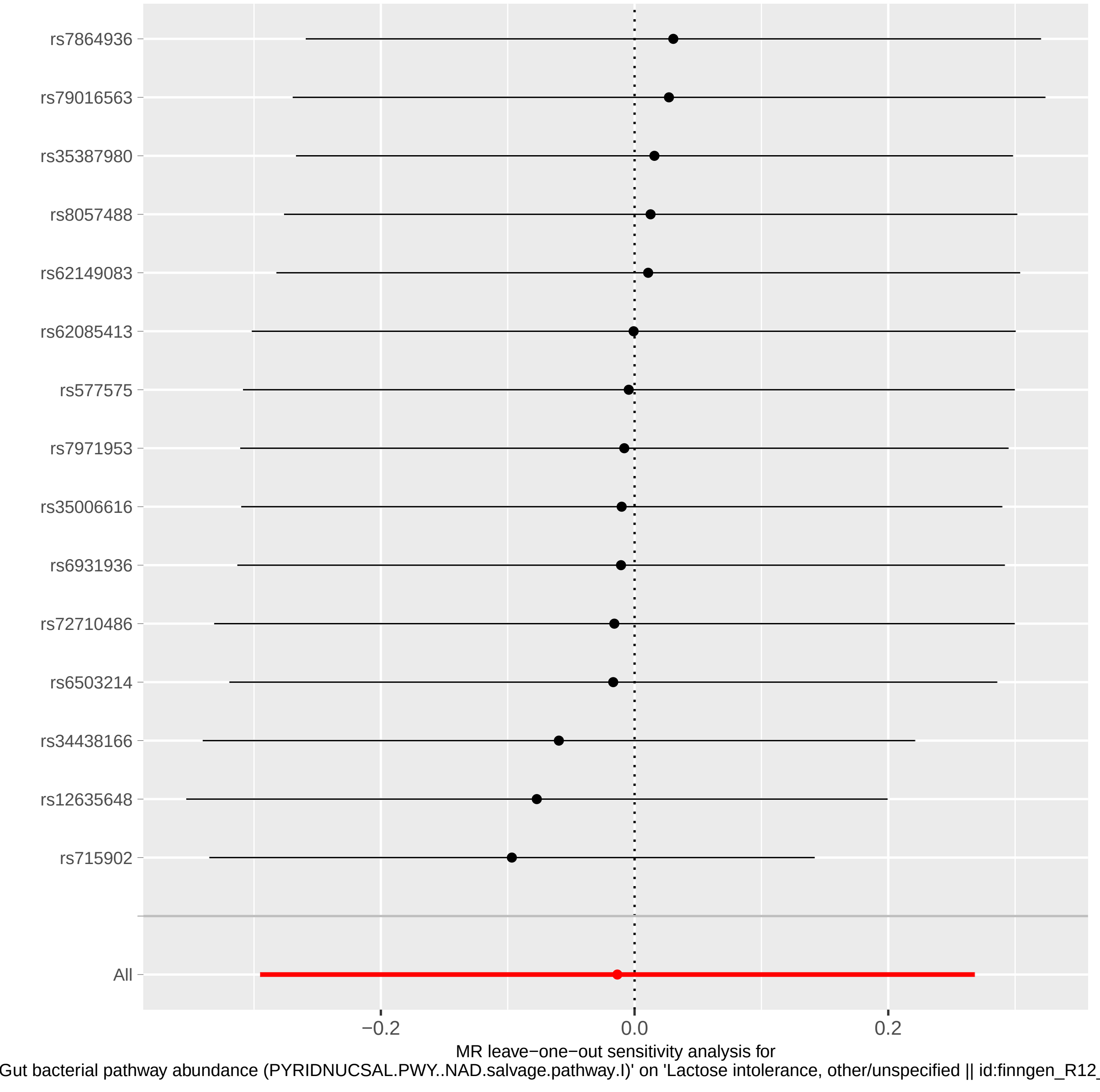
rs7864936
rs79016563
rs35387980
rs8057488
rs62149083
rs62085413
rs577575
rs7971953
rs35006616
rs6931936
rs72710486
rs6503214
rs34438166
rs12635648
rs715902
All
−0.2
0.0
0.2
MR leave−one−out sensitivity analysis for
Gut bacterial pathway abundance (PYRIDNUCSAL.PWY..NAD.salvage.pathway.I)' on 'Lactose intolerance, other/unspecified || id:finngen_R12_
